# Impact of sex differences in adult and paediatric HIV-1 cure

**DOI:** 10.1097/COH.0000000000001038

**Published:** 2026-05-15

**Authors:** Maximilian Muenchhoff, Nomonde Bengu, Philip J.R. Goulder

**Affiliations:** a Max Von Pettenkofer Institute and Gene Center, Virology, National Reference Center for Retroviruses, LMU München; b German Center for Infection Research (DZIF), Partner Site, Munich, Germany; c HIV-1 Pathogenesis Programme, Doris Duke Medical Research Institute, Nelson R. Mandela School of Medicine, University of KwaZulu-Natal; d Africa Health Research Institute, Durban, South Africa; e Peter Medawar Building for Pathogen Research, Department of Paediatrics, University of Oxford, Oxford, UK; f Ragon Institute of Massachusetts General Hospital, Massachusetts Institute of Technology and Harvard University, Cambridge, Massachusetts, USA

**Keywords:** immune sex differences, natural killer cells, natural killer cells, paediatric HIV, type I interferon

## Abstract

Stronger type I interferon responses in females compared to males, starting from intrauterine life, underpin the sex differences observed in HIV-1 cure/remission outcomes in children and adults. In adults these innate immune sex differences favour females achieving HIV-1 cure/remission over males. Recent studies of the adult viral reservoir reflect the ability of innate immune responses in females, including natural killer (NK) cell activity, to remove cells harbouring intact proviral DNA more effectively than males. In children, the situation is more complex. Initially, in the first years of life, males have a higher propensity to achieve HIV-1 cure/remission, at this stage benefiting from the effects of having weaker interferon (IFN)-I responses, including low baseline HIV-1 DNA loads and being recipients of an IFN-I sensitive transmitted founder virus. By mid-childhood, the picture is mixed, with the impact of stronger innate immunity in females combined with the development of more effective HIV-specific CD8^+^ T-cell response via immune ontogeny tending to favour females beyond the age of 5–10 years. In children, therefore, the double-edged sword effects of IFN-I in the setting of vertical transmission and immune ontogeny results in distinct, dynamic sex advantages through childhood.

## INTRODUCTION

The impact of immune sex differences on HIV-1 disease outcome in antiretroviral therapy (ART)-naïve adults was not appreciated for some considerable time into the pandemic. The initial observations that initial viral loads are lower in women, but that disease progression for a given viral load is faster in women [1,2], were made more than 15 years after the initial cases were described [3] in 1981. The landmark paper by Meier *et al.*[4] which showed that plasmacytoid dendritic cells (pDCs) from women produce more interferon (IFN)-α than pDCs from men when stimulated by TLR7 ligands such as single stranded RNA from viruses such as HIV-1, highlighted the double-edged sword of immune activation: low viral setpoint requires rapid activation of the immune system [5], but immune activation also ‘fuels the fire’ of viral replication [6], thereby accelerating disease progression. [7]

It was >35 years into the pandemic until it was appreciated that women are as much as fivefold more likely to achieve ‘elite’ control of HIV-1 infection [6]. Indeed, black females are 10-fold more likely to achieve elite control than white males [6]. Despite the dramatic impact of sex and ethnicity, these differences in HIV-1 outcomes were slow to emerge because most studies were focused on either B clade-infected Caucasian MSMs or C clade-infected African women. Direct comparisons of males and females were therefore confounded by differences in HIV-1 clade and ethnicity. An illustration of this was the SPARTAC Trial [8], which included female participants from South Africa and Uganda (non-B clade virus); and non-African male participants from Europe, Australia and Brazil, (B-clade virus). Time to viral rebound following treatment interruption was approximately 10-fold longer in the African, female, C clade-infected participants (*P* < 0.001) [9]. Despite the confounding influences of clade and ethnicity, this was a hint that, in adult infection, females have a greater propensity to achieve cure/remission than adults.

By contrast, comparisons of sex differences in HIV outcomes for children matched for ethnicity and clade of infection, are easier to conduct. However, the impact of sex differences in children are more complex because they change through childhood. For example, HIV-1 plasma viral loads are initially lower in males [10,11], but after 2–3 years of life, HIV-1 plasma viral loads are typically lower in females [12], as in adults [1,2]. In part, this complexity is result of the timing of hormonal changes, with gonadal hormones stimulated via activation of the hypothalamic–pituitary–gonadal axis in three distinct waves: *in utero* (at 8–24 weeks’ gestation), in infancy (‘minipuberty’, at 1–6 m of age) and in puberty [13,14]. Also, these changes over time result from immune ontogeny, with early-life responses characterised by highly regulated, tolerogenic immunity and notably weak support for Th1-driven immune activity until mid-childhood and adolescence [15,16]. For this reason, immune control of HIV-1 in the first few years of life in ART-naïve children depends more on innate immunity, notably via antiviral natural killer (NK) cell responses, than on HIV-specific CD8^+^ T-cell activity, whilst in ART-naïve adults the reverse is the case [17,18].

In this review, we will reflect on the recent findings in relation to immune sex differences, and how these may influence the ability of adults and children to achieve HIV cure/remission. In this review, references to “males” and “females” denote biological sex based on genetic makeup. This distinction emphasizes innate sex differences rather than gender identity or social constructs. 

**Box 1:**
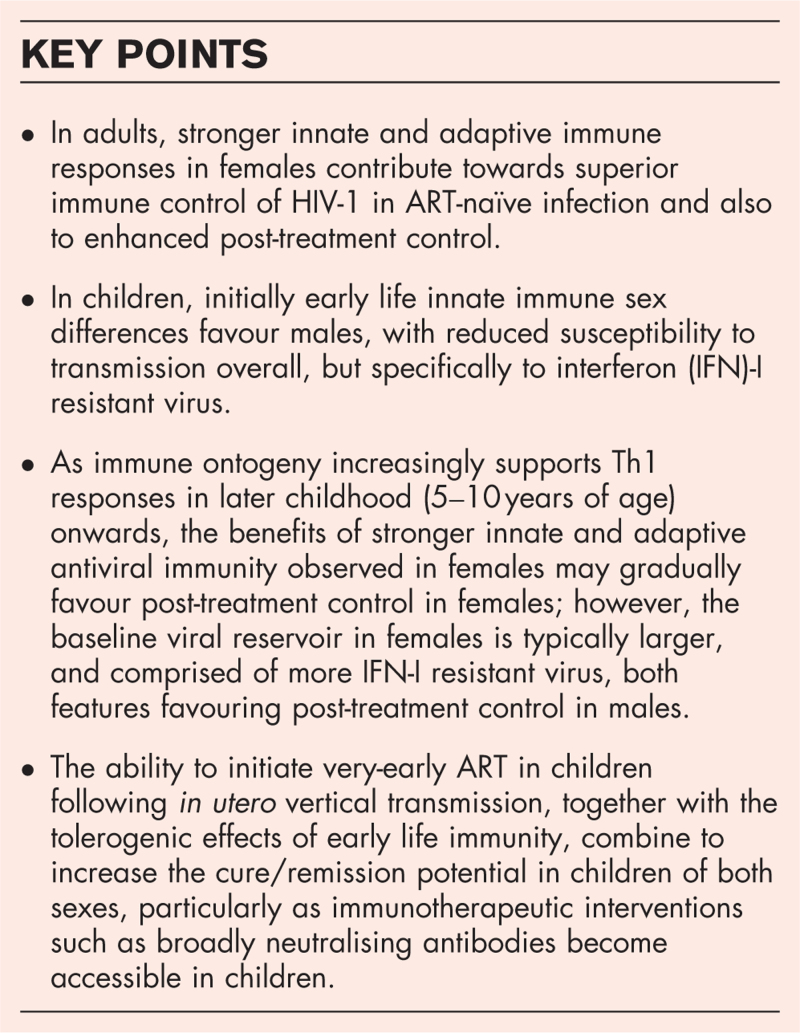
no caption available

## FEMALE ADVANTAGE IN ADULT CURE OUTCOMES

The three principal mechanisms underlying immune sex differences are inter-related: genetics, epigenetics and sex steroids. The increased type I interferon (IFN-I) production by pDCs from females in response to stimulation by TLR7 agonists, observed in several studies [4,19–22], that is fundamental to activation of innate and also adaptive antiviral immunity, results from escape from X chromosome inactivation (XCI) of the *TLR7* gene, such that approximately 40% of innate immune cells in females are bi-allelic for *TLR7*[23]. The benefits of increased IFN-I production in ART-naïve HIV-1 adult infection is associated with the 0.35–0.5 log_10_ lower viral setpoints in females compared to males [24]. The notorious double-edged sword of the IFN-I response is well-illustrated by the study of *TLR7* variants by Azar *et al.*[25]: the common polymorphism reduces TLR7 abundance but in homozygotes is associated with 0.85 log_10_*lower* viral setpoints. This suggests that the relationship between IFN-I responses and immune control of HIV-1 is a U-shaped curve: increased IFN-I production is beneficial in enhancing immune control of HIV-1 up to a point, but beyond that point the detrimental effects of inflammation outweigh the advantages. [23]

A second X-linked gene which partially escapes XCI and plays an important part in immune sex differences is *Kdm6a,* which encodes the protein UTX (ubiquitously transcribed tetratricopeptide repeat on chromosome X), a histone demethylase and epigenetic regulator which has a critical influence on the effectiveness of NK responses and is the reason why NK cells are lower in number but more effective in females [26]. Mice with a homozygous deletion of this gene fail to survive challenge with a sublethal dose of MCMV. Humans with NK cell defects are highly susceptible to CMV and other herpesvirus infections [27]. CMV coinfection results in higher rates of HIV-1 disease progression in both children and adults LWH [28,29]. Unexpectedly, early CMV acquisition appears to be highly detrimental to HIV-exposed uninfected male infants born to mothers LWH. In a recent study of Zimbabwean children, mortality over the first 18 months of life was increased sixfold in males whose mothers were CMV viraemic in pregnancy [30]. This effect was not present in males born to HIV-negative mothers and was not significant in HIV-exposed uninfected females whose mothers were CMV viraemic in pregnancy. Together these data suggest the hypothesis that CMV coinfection in children LWH, and possibly also adults LWH, may have a detrimental impact on HIV cure/remission potential in males as a result of inferior NK-mediated control of CMV and other common herpesvirus infections, and consequently higher levels of immune activation and inflammation.

The impact of sex steroids on immune function and specifically on HIV-1 cure/remission has recently been demonstrated by studies of trans men receiving gender affirming testosterone [22,31]. The effect of testosterone on the immune system, often categorised as being immunosuppressive, is more nuanced, favouring production of tumour necrosis factor (TNF)-α rather than IFN-I by pDCs and monocytes, increased TNF-α signalling via NF-κB in innate immune cells and enhanced IFN-γ responses in NK cells [31]. Longitudinal studies of trans men receiving gender affirming testosterone show the reduction in IFN-I production by pDCs from levels in cis women to the same levels as in cis men over a period of 42 weeks [22]. Interestingly, pDC cytokine responses in response to TLR7 stimulation in cisgender women LWH stably suppressed on ART negatively correlate with levels of intact proviral DNA [32], further supporting the notion that testosterone has a negative impact on intact viral reservoir size and therefore on cure potential in adults.

Further evidence in adults that sex steroids may play a significant impact on the viral reservoir came from a study that identified estrogen receptor-1 (ESR-1) as a key factor regulating HIV-1 latency [33]. Selective estrogen receptor modulators activated latent proviruses in males and females whereas β-estradiol inhibited reactivity. This study and others [34] have shown that females have lower inducible reservoirs compared to men. However, in males and females aged approximately 45–55 years, total HIV-1 DNA declines more slowly in women than in men, and the inducible HIV-1 reservoir actually increases in women after menopause [35]. These data suggest that, over the reproductive lifespan, estrogens such as β-estradiol enhance viral latency.

That females may have a greater chance of achieving cure/remission than males is further supported by case reports of potential sterilising cure in 3 female elite controllers. Among EC, the total proviral HIV-1 DNA load is usually detectable at approximately 1 copy per million PBMC, and the intact HIV-1 proviral DNA load at approximately 1 in 10^7^ PBMC. In two cases who underwent leukapheresis, the SF patient and the Esperanza patient, total DNA was present at 0.6 and 1.3 copies per 10^8^ PBMC, respectively, and no intact proviral DNA was detected in >1 × 10^9^ PBMC analysed [36,37]. More recently, a third female case of potential sterilising cure was described, again in whom no intact proviral DNA was detected. However, in this instance, leukapheresis was not possible, thus limiting the number of PBMC available for analysis [38].

In recent, more direct comparisons of the male and female viral reservoir, Tan *et al.*[39^▪▪^] studied 65 adults who had been virally suppressed for 20 years. Although total and intact proviral DNA loads declined over time in males and females, there was no sex difference observed in total or intact HIV-1 DNA load. However, there were clear qualitative sex differences in the viral reservoir. In females, there was a greater number of clonally expanded intact proviruses, and a higher proportion of intact proviruses integrated into repressive heterochromatin locations. This finding implies stronger immune selection pressure in females for intact proviruses to be located in regions of the genome that limit the ability of the immune system to recognise and remove cells harbouring intact HIV-1 proviral DNA. The authors identified evidence that the innate immune system, specifically NK cell responses, may be mediating this sex-specific immune selection pressure on the viral reservoir. By contrast, in males, HIV-specific T-cell responses are principally mediating removal of cells harbouring intact provirus [39^▪▪^].

Together these studies highlight the dynamic nature of the viral reservoir, reducing in size with increasing duration on ART but also changing qualitatively, with sex differences increasingly apparent over time as the differential impact of male and female immunity imposes sex-specific selection pressure on the cells harbouring intact proviral DNA. To date, no studies of sex differences within the paediatric viral reservoir have been undertaken.

These studies on sex differences in the viral reservoir in adults are also consistent with the broad findings from studies of post-treatment or post-intervention controllers, which often have not involved female participants, where the features identified with increased time to viral rebound following treatment interruption are: early ART initiation following acquisition of HIV-1, and therefore a small and phylogenetically narrow viral reservoir [40,41]; low levels of immune activation, and absence of immune exhaustion markers [42,43]; strong IFN-I responses pre-ATI [44]; robust NK responses [45–47]; stemlike HIV-specific CD8^+^ T-cell responses [48,49,50^▪^,51^▪^].

To this list may be added disease-susceptible HLA class I molecules, such as HLA-B*35 in B clade infection and HLA-B*18/B*45/B*58:02 in C clade infection. A striking finding from the VISCONTI study [42,47] and other cohorts [52] is the paradoxical low frequency in post-treatment controllers of ‘protective’ HLA-I such as HLA-B*57, and high frequency of disease-susceptible HLA-I. However, the impact of ‘protective’ and ‘disease-susceptible’ HLA on time to viral rebound following treatment interruption may be affected by timing of ART initiation and interventions such as broadly neutralizing antibodies (bnAb) therapy. In the FRESH cohort trial participants, for example, who mostly initiated ART during Fiebig I, longer time to rebound and lower viral setpoints during ATI following intervention with bnAbs and a TLR7 agonist were associated with protective HLA [54]. Additionally, it is possible that sex may influence the impact of ‘protective’ and ‘disease-susceptible’ HLA on cure outcomes: for example, all FRESH study participants were female. [53]

## DYNAMIC IMPACT OF SEX ON CURE OUTCOMES IN CHILDREN

Studies of the transmitted virus have highlighted some major differences between paediatric and adult transmission. In adults, the numbers have not been studied to date to compare the nature of viruses in male-to-female, female-to-male and male-to-male transmission. However, from detailed studies of 8 adult transmission pairs [55], which included examples of male-to-female, female-to-male and male-to-male transmission, in all these scenarios there is strong selection of an IFN-I resistant virus being transmitted [55–57].

Equivalent studies have been undertaken in 98 mother-child pairs following *in utero* transmission [10]. These evaluations employed the Gag-Pro/NL4-3 chimeric virus approach [58], in which *gag-pro* from each study participant was amplified and cloned into an NL4-3 backbone. The advantage of this high-throughput approach is that it is less labour-intensive than analysis of 20-30 near full-length viral genomes per transmission pair, and therefore can be employed to evaluate large study numbers. In addition, the polyclonal amplification of the swarm of viruses present in the sample exactly reflects the circulating swarm of viruses, whereas it is more difficult to demonstrate that a small number of viral clones is representative of the quasispecies.

Striking differences were observed between vertical transmission arising in mothers with chronic infection (mothers whose diagnosis was known prior to the pregnancy) compared with those with ‘recent’ infection - mothers who either seroconverted during pregnancy, or whose diagnosis was first made during the pregnancy. In adults [59], the transmitted IFN-I resistant virus is overgrown by IFN-I sensitive virus over the course of the first 6–12 months of infection. This finding was confirmed in the mother-child transmission study, with the chronically-infected mothers harbouring IFN-I-sensitive virus and the recently-infected mothers principally harbouring IFN-I-resistant virus. By contrast with adult transmission, the virus transmitted by mothers with chronic infection was IFN-I sensitive. There was no significant selection of vertically transmitted virus on the basis of IFN-I IC50. In the case of mothers with recent infection, the IFN-I resistant virus that was circulating in the mothers was the same IFN-I resistant virus that was transmitted; again, there was no selection of vertically transmitted virus on the basis of IFN-I IC50. However, whereas female foetuses were susceptible to IFN-I resistant viruses, male foetuses were not.

**FIGURE 1:**
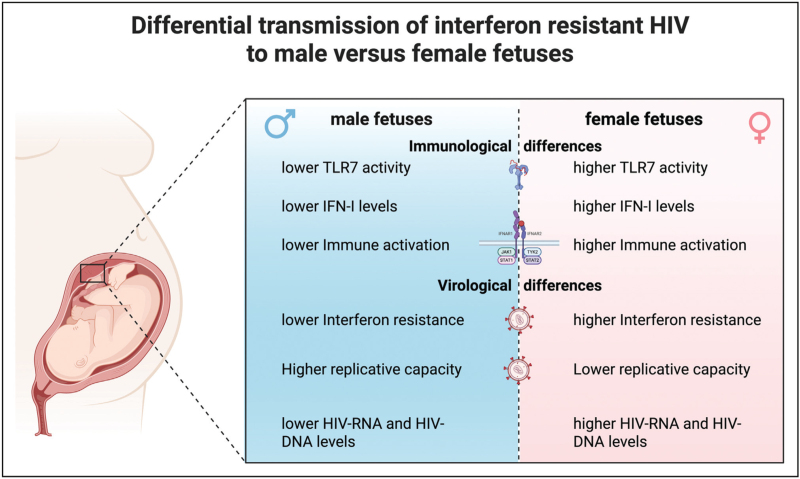
Summary of immunologic and virologic features favouring transmission of IFN-I resistant viruses to female versus male foetuses.

**FIGURE 2:**
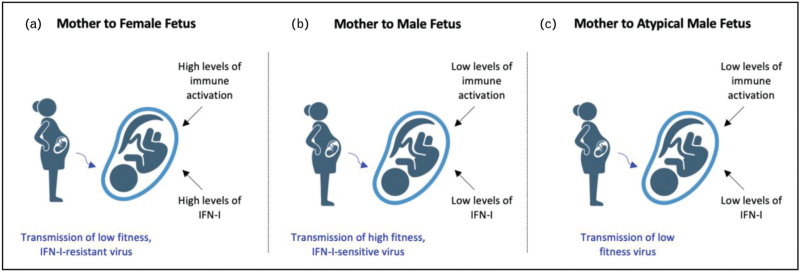
Distinguishing features of immunologic and virologic features favouring HIV-1 cure/remission in males versus females. (a) Mother to female foetus: typically, the virus transmitted is low ‘fitness’, IFN-I resistant. (b) Mother to male foetus: typically, the virus transmitted is high ‘fitness’, IFN-I sensitive. (c) Mother to ‘atypical’ male foetus who achieves cure/remission: low ‘fitness’ virus is transmitted, possibly related to the generation of rare variants in the setting of high immune activation during recent acute HIV-1 infection in the mother.

In part these sex differences in susceptibility to IFN-I resistant viruses may be related to the fact that IFN-I resistant vertically transmitted viruses tend to have low viral replicative capacity [10]. Whereas low replicative capacity viruses can be transmitted and flourish in female foetuses and infants, this appears not to be the case for males, who are principally susceptible to IFN-I sensitive viruses with high replicative capacity. In rare cases where low replicative capacity viruses were successfully transmitted, several of these children achieved sustained aviraemia following unscheduled treatment interruption or, in the Azaphile trial, following ATI [60] (Figs. 1 and 2).

The mechanism underlying these intra-uterine sex differences in HIV-1 susceptibility is likely related to the stronger IFN-I innate immune responses in females of all ages, from foetal life through childhood, adolescence and into adulthood [4,19–22]. The significance of these early life innate immune sex differences is that, first, females are more susceptible to *in utero* transmission in the setting of recent maternal infection, when circulating maternal viruses are IFN-I resistant; and second, as discussed further below, cure potential is higher, at least initially, among males following vertical transmission.

These findings in relation to vertical transmission are consistent with adult studies of sex differences in a heterosexual Zambian transmission cohort [61]. In that study, where viral replicative capacity (‘fitness’) was inferred from analysis of viral sequences alone, the authors observed that lower fitness viruses within the swarm tended to be transmitted from males to females, whereas higher fitness viruses within the swarm tended to be transmitted from females to males. The conclusion, that female adults are biologically more susceptible to HIV-1 transmission than males, is consistent with the observations of greater female susceptibility in vertical transmission [10,62–66], and also with the observations of a 3.8:1 female:male sex ratio in new infections in adolescents [67].

The reasons why male children, at least initially, have a better chance of cure/remission are related to the early life innate immune sex differences described above. First, the mothers who transmit to males carry IFN-I sensitive virus. Plasma viral load in these mothers correlates with viral IFN-I resistance [10], so mothers of males harbouring IFN-I sensitive virus tend to have low viral loads. Low plasma viral load in mothers of males in itself may limit the inoculum and number of viral variants transmitted [68] to males. In addition, as mentioned above, the higher levels of immune activation in female foetuses [62] may also contribute to the ~0.5 log_10_ higher baseline plasma viral loads, and total HIV-1 DNA loads in females versus males [10,69]. Second, studies in adults [59] and in children within the Azaphile trial [60] show that rebounding virus following ATI is highly IFN-I resistant, and, in adults, even more so than the IFN-I resistant transmitted founder virus. This implies that IFN-I sensitive viruses can be contained by IFN-I responses mediated via innate immune cells in the lymphoid tissues. Therefore, an initial viral reservoir constituted of IFN-I resistant virus, which is typically observed in females and not in male children, is by definition pre-adapted to evading such innate immune control.

As stated above, in children >2–3 years old [11,12], immune control of HIV-1 viraemia in ART-naïve children is superior in females. Immune control of HIV-1 in early life is mediated predominantly through NK cell responses rather than HIV-specific CD8^+^ T-cell responses [17], but increasingly towards the latter part of childhood (ages 5–10 years), HIV-specific CD8^+^ T-cell responses play the dominant antiviral role [70,71], as in adults. Since the advent of ART it has been difficult to evaluate HIV-specific CD8^+^ T-cell responses, especially in children when ART is initiated at birth. However, it is clear from historical studies [70,71] that, even in ART-naïve children who maintain normal-for-age CD4^+^ T-cell numbers, HIV-specific CD8^+^ T-cell responses are of very low magnitude and breadth in the first 5 years of life. As Th1 responses are increasingly supported through the latter period of childhood from 5–10 years of age, the impact of stronger IFN-I responses in females in activating the HIV-specific CD8^+^ T-cell component of the anti-HIV-1 response is a major factor underpinning the superior control of viraemia in female children aged 5–10 years, adolescents and adults. Elite controllers in children are much rarer than in adults, and indeed to date there is only a single study published on paediatric elite controllers [72]. In that study of 11 elite controller children, all but one but were females.

Thus, in children aged >5 years, many of the factors favouring an increased propensity of adult females compared to males to achieve HIV-1 cure/remission apply. The main difference however may relate to the ‘head-start’ that male children have at transmission and in the first 2–3 years of life, as a result of the benefits of weaker innate immune responses, transmission of IFN-I sensitive virus and a smaller initial viral reservoir.

## EVOLUTIONARY BASIS FOR IMMUNE SEXUAL DIMORPHISM

These studies of immune sex differences highlight their critical importance in HIV-1 cure/remission outcomes but also serve as a reminder of their impact across the broad range of infectious diseases, inflammatory conditions, and cancers [73,74]. Furthermore, this influence starts *in utero*[10] and operates across the entire lifespan [73–75]. The pattern that emerges is of greater investment in the immune response by females, and consequently more robust immune responses, and superior outcomes in terms of morbidity and longevity. The double-edged sword of IFN-I responses is well illustrated by the higher levels of autoimmune diseases and serious adverse events to immunizations typically seen more often in females [73,74]. In this review, we have seen that, in adults, a stronger IFN-I in females results in lower viral setpoints but more rapid CD4 decline and disease progression for a given viral setpoint than in males, and that small changes in the IFN-I response can substantially outcomes [25]. In vertical transmission, higher immune activation resulting from stronger IFN-I responses in female foetuses results in increased susceptibility to vertical transmission, specifically to IFN-I resistant viruses. Male foetuses are less susceptible altogether to vertical transmission than females, and the fact that male foetuses are susceptible *only* to IFN-I sensitive virus is an advantage in achieving cure/remission. As children progress towards later childhood (5–10 years of age), the advantages of stronger IFN-I responses in females, together with the effects of immune ontogeny, with an increasing role for HIV-specific CD8+ T-cell activity, enhance the prospects of HIV- cure/remission in females of this age.

The observed increased investment in immunity made by females versus males prompts the question of its evolutionary basis. Darwinian sexual selection describes within-specifies sex differences in appearance and behaviour that result from the trade-off between reproductive success and survival [76]. A case in point is the male peacock whose ornamental secondary tail feathers enhance reproductive advantage [77] at the cost of survival disadvantage, and this trait therefore could not be explained by the theory of natural selection alone [78]. The starting point for these sex-specific, divergent, evolutionary forces driving this process is anisogamy [76], the unequal size of male and female gametes. The result of anisogamy is that males can produce many more gametes than females, and there is potentially much more variance in the reproductive success of males than that of females. Consequently, selection pressure is stronger on males for traits that increase the chances of reproductive success, such as weaponary and size to ward off competing males; and ornamentation and courtship displays to attract females [79]. Given that resources are limited, successful males have invested in these traits that maximise reproductive success, at the cost of longevity. By contrast, for females, resource allocation favouring longevity, and therefore investment in a robust immune response, also favours reproductive success. Greater female investment in their offspring means that enhanced immunity in females also improves survival of their children. No clearer example of this can be found in HIV-1, where mortality in infants of mothers LWH with absolute CD4 counts of <200 cells/mm^3^ was increased more than threefold, irrespective of whether the child was HIV-infected [80].

## CONCLUSION

In conclusion, the impact of immune sex differences on cure outcomes is more complex in children than in adults, but the influences of very early ART combined with tolerogenic early-life immunity present opportunities to achieve cure in children that are not so easily available in adult infection. The impact of sex differences in adults and children on cure outcomes is summarised in Table 1. In the Azaphile trial, 32% of very-early ART-treated children with low-to-undetectable total HIV-1 DNA loads achieved cure remission, with a median time to viral rebound of >18 months [60]. As interventions, particular broadly neutralising antibodies, become more accessible in paediatric studies, it is reasonable to speculate that the chances of achieving significant levels of HIV-1 cure both in female and male children may be enhanced substantially.

**Table 1:** Summary of the sex differences in factors related to adult and paediatric HIV-1 cure

	Adults	Children
Principal routes of transmission	Horizontal: M-to-F, F-to-M, MSM	Vertical: mother-to-M, Mother-to-F
Transmitted virus	IFN-I resistant, high fitness (M>F)	Males: IFN-I sensitive, high fitness Females: IFN-I resistant, low fitness
Susceptibility to HIV-1 transmission	F>M	F>M
Plasma viral RNA setpoint	F<M 0.35-0.5 log_10_	Birth-2yrs: M<F 0.5 log_10_ >3yrs: F<M
Elite control (EC)	EC 5x F>M; potential sterilizing cures among ECs: all 3/3 female	EC 10x F>M
Immunity: innate, NK	IFN-I responses: F>M NK cell responses: F>M	IFN-I responses F>M
Viral reservoir	Total and intact: F≤M Qualitative sex differences, NK-driven selection on reservoir F>M	Total HIV-1 DNA: 0.5 log_10_ M<F at birth
Sex steroids	Testosterone reduces IFN-I production by pDCs (trans males); increased IFN-I production → low intact proviral load (cis females); estrogens e.g. β-estradiol increases viral latency F>M	No data in children

The table summarises the published data cited in the text.

## Acknowledgements

*This work is supported by grants to P.G. from the National Institutes of Health (UM1-AI164566, Pediatric Adolescent Virus Elimination (PAVE) Martin Delaney Collaboratory; and U01-AI168655).*

### Financial support and sponsorship

*None.*

### Conflicts of interest

*There are no conflicts of interest.*

## REFERENCES AND RECOMMENDED READING

Papers of particular interest, published within the annual period of review, have been highlighted as:

▪ of special interest
▪▪ of outstanding interest
